# A Suggestion to Christendom

**Published:** 1855-06

**Authors:** 


### HALL'S JOURNAL OF HEALTH.

OUR LEGITIMATE SCOPE IS ALMOST BOUNDLESS: FOR WHATEVER BEGETS PLEASURABLE AND HARMLESS FEELINGS, PROMOTES HEALTH; AND WHATEVER INDUCES DISAGREEABLE SENSATIONS, ENGENDERS DISEASE.

VOL. II.] JUNE, 1855. [NO. VI.

### A SUGGESTION TO CHRISTENDOM.

TAKING it for granted that the Bible is a revelation of Heaven, whose object is to Christianize the whole human family: that this should be done in the speediest manner possible: that it is not to be accomplished by miraculous power, but through human instrumentalities, the main of which are the circulation of the Scriptures of the Old and New Testaments, and their oral exposition by educated men: that "*the field is the world*," embracing every kingdom, and tribe, and people of the habitable globe, and assuming the conceded fact, that multitudes are daily passing down to endless night, for the lack of that gospel; admitting these things to be true, what is the obvious, the great practical inference which obtrudes itself, with resistless power, on the intellect and the heart of every thoughtful Christian?—That whole armies of educated ministers should be starting out from the church all the time, bound for all the world, with copies of the Sacred Scriptures as numerous as the drops of morning dew, for *judicious* distribution.

How to raise these armies of new clergymen, I purpose discussing, one of these days, with a startling, yet truthful development, not seen by the many now, but by the few seen and winked at.

The point most deplored at present, is the fewness of the laborers, and the alarming fact, as to the United States at least, that ministers are but slowly increasing as to numbers and population, while there are fewer communicants, proportioned to the whole people, than there was eleven years ago; while foreign immigration, with its anti-christian, revolutionary and pantheistic sentiments, with its indecent and impertinent interference with our institutions, loudly demands, in tones of a thousand thunders, *the immediate and large increase of a thoroughly educated and well paid ministry.*

But next in importance to increasing the number of clergymen, is the taking proper care of those who are already in the field; and this brings us within the more immediate scope of a Journal of Health. I mean the preservation and restoration of the health of ministers. As a class, I think I may very truly say, the clergy of the United States are a sickly race. You can scarcely come across one anywhere, who has not an ache, or a pain, or a symptom of some kind; and it is notorious, that many of them perish long before their prime, or are rendered incapable by ill-health of continuing their official duties, and thus their services are almost wholly lost to the church, as far as their ministerial functions are concerned.

As to those who are blest with good health, they may be dismissed with the suggestion that it is their duty, as it is in their power, to maintain that good health; that it is a talent entrusted to their care, and for its preservation and employment, the great Head of the church will hold them to a strict accountability. To the large class of invalid clergymen, and to the church, whose servants they are, I wish to make some suggestions, which would give a vigor to church aggression, to "*church extension*," which no man or set of men now living have perhaps dreamed of.

Hunt up every regularly educated sick clergyman in the land, who is able to mount a horse, and whose standing among his brethren is "*without a spot or wrinkle, or any such thing;*" assure him of two dollars a day, rain or shine, throughout the year, regularly paid on the last day of each month, present him with a good horse, a Bible, a hymn book, and a concordance, adding, if you please, the prayer book, or a confession of faith of his denomination, and require him to travel and preach every day and Sunday too, continuous, from one year's end to another, reserving his daily wages, and imposing a fine of two dollars for every day he does not preach; require him to preach at least four times a year at each post-office station within a certain number of counties; let him be required not to sleep in the same house over twice a year, nor to eat at the same table more than thrice in succession. Now, what would be the effect of a plan like this, carried out with energy ; such an energy, indeed, as the times of the world now most imperatively demand? Some of the first and more immediate results would be, that

1. A large class of our worthiest, best educated, law-abiding, most influential, and competent men, would be relieved of that crushing apprehension of want, which is enough to cripple the energies of the stoutest heart.

2. The gospel, as to its great essentials, would be preached to thousands every day; who do not now hear it, in its purity, once a year, or even in a decade, in places not a few, even on this side the Mississippi River, as I know.

3. From the very first week, the health of these men would improve, in nine cases out of ten, while the ease of preaching, being a daily business, would steadily increase, whether they had Bronchitis, Throatitis, Lungitis, or any other kind of "itis,"

4. By eating and sleeping at different houses every time, they would have opportunities of offering a prayer here, of reading a chapter there, and dropping a word of exhortation yonder, to some diffident boy, some shamefaced girl, some rough farm-laborer or neglected servant maid, which *might*, yes! would, with demonstrable certainty, spring up afterwards to the fruitage of a Webster, a Catharine, or a Joseph Hume. How are the watchmen sleeping, while the day almost breaketh, the time for their labor almost gone!

Be assured, Christian men and women, the Sebastopol of the Prince of darkness will never be taken, as long as you and your ministers work but one day in seven. If I understand aright, you enlisted "*for the war*," not for one-seventh portion of it. Religion and preaching must be an every day work to every one of you, or the millennial day glory will never dawn on your eyes; especially while so many of your leaders, your clergy, are on the sick list. There is full, there is double work for every one of them; the church cannot afford to have a single hand sick; wake up, then, to some such plan as I have now suggested, and its heaven-saving effects will not cease, till the last wave of time is rolled into eternity's ocean.

These are not mere sounding words, the jingle of a well-turned sentence, but a great truth, that work banishes sickness, that hard, steady labor promotes sound health, *with constantly increasing capabilities of mind and body*, while such an entire immersion in soul-saving, as every day preaching would involve, would in time give a presence, a moral power to the speaker in every emotion of his heart, in every glance of his eye, in every expression of his countenance, almost equal to the eloquence of an angel. It was the brightness of Moses' face, when he had been forty days in communion with his God, which carried with it a power, almost supernatural, over the hitherto impenetrable hearts and consciences of the Israelitish multitude.

As confirmation of some of the statements made, I give here the illustration of facts in the case of Dr. Williams, who is even now preaching with almost youthful vigor, and with more than youthful efficiency, three times every Sabbath day, at the age of seventy-five years. Fie upon you, ye sickly folk, yet in your twenties, whom a single Sunday's discourse "*lays up*" for the balance of the week; go to, crack rock, peddle books on commission, do anything honorable to secure the means of purchasing a horse, if your Christian brethren around you are not noble-hearted enough to furnish you, and like good old Father Williams, scale mountains, penetrate valleys, ride miles by the thousand, and by it get the health which he has, and which these very same things helped to secure to him, and like him live in usefulness, and efficiency, and honor, to the same green old age. But start not out, unless the minimum salary I have named is guaranteed by the church, whose servant you are; on the ground that *the laborer is worthy of his hire.* A common drayman gets two dollars a day, and surely an educated clergyman is worth a drayman's wages, when it is considered that his mission is not to lift up bales and boxes, but to raise up men; not to transport bags and barrels along the highway, but to conduct humanity to heaven. If the church will not sustain you, then you are free to lay down your tools, as her public servant, and to shake the dust from your feet as her disgrace and her anathema. Working for nothing and finding oneself is not the requisition of the Christian system; a man may do it, but his Master does not require it of him; no man of reflection can possibly entertain such a sentiment for a moment, if considered maturely.

The point I am arriving at is the restoration to full health and to capabilities of full ministerial duty of the large number of clergymen, who are now more or less inefficient in consequence of ill-health; of health lost in the service of the church. A soldier disabled in trying to shoot his brother man, is at once placed on the pension list, but a clergyman whose youth and manhood have been spent in the effort to throw around this world a chain of love, and raise it up to heaven, if he is disabled in his God-like work, is turned out to die like an old dray horse.

The first step towards their regeneration is to place them and their families above the fear of want; a second means is to present to their eyes an open door of labor in the good old cause, around which gather the fond associations of youth departed; even like the old war-horse, the first sound of the trumpet "to charge," will of itself awaken them out of half their ailments; for be assured, that a man who has been once a clergyman at heart, will never cease to be one in will, whenever there is a way.

These two items—relief from the fear of want, by the securement of a liberal salary, and the prospect of immediately entering upon their favorite work,—would at once produce such a reaction in the nervous system and that of the blood, as would make of this estimable class, new men; while being followed up by the healthful influences of horse-back exercise, of out door air, of change of food, and of a new object ahead, some new point to be gained, every hour, there would be a change of bodily health for the better, which, if any medicine could produce, would give its possessor, in a single year, the wealth of Crœsus a thousand times told. As confirmatory of these views, and as food for serious consideration, I will close this,. without further remark just now, with a communication from the ably conducted Baptist paper, the *Watchman and Reflector*, Boston.

"The reverend and venerable Dr. Williams is closely connected with the family of John Henry, who in 1693 suffered martyrdom in London, and is considered by many as the "morning star of the reformation of Cambria," and the originator of the first proposal of the movement of the Pilgrim fathers to this country. Mr. Williams inherits no small portion of the noble spirit of the martyr. He is pastor of the Independent or Congregational church at Troedrhindalar, in Wales, and for fifty years has maintained a high reputation throughout his native country as a preacher and pastor, and is yet a hale and vigorous workman. He is the oldest minister in Wales, and though now seventy-five years of age, preaches regularly three times every Lord's day. He began to preach when he was twenty-one; was ordained a pastor in 1803, and for some years his labors were of the most arduous and self-denying character; and though his engagements have been varied and laborious, and have compelled him to take frequent journeys across the mountains, and almost pathless wilds, through rain and snow, he has never once been disabled from preaching. In his younger days he was considered a splendid horseman, and even now he would tire many a hunter. He must have spent some years in the saddle, for the number of miles he has traveled on horseback is almost incredible. As the apostle of Breconshire, and as a regular preacher for forty years, at all the large out-of-door gatherings in North and South Wales, his traveling has been beyond reckoning.

The church at Troedrhindalar, of which he is pastor, has had only three ministers during the last one hundred and sixty years. The present pastor has held his office for fifty years; his predecessor, the Rev. Isaac Price, was the minister for fifty years; and his predecessor, the Rev. Thomas Morgan, was the minister for sixty years. Mr. Williams testifies that during the whole of these one hundred and sixty years, the church has enjoyed uninterrupted peace and harmony. During his pastorate alone it has received above fifteen hundred into fellowship.

